# Tobacco use and sleep loss over worry among adolescents aged 12-15 years: A population-based study of 38 countries

**DOI:** 10.7189/jogh.10.020427

**Published:** 2020-12

**Authors:** Qian Wang

**Affiliations:** School of Public Health, Shanghai Jiao Tong University School of Medicine, Shanghai, China

## Abstract

**Background:**

Sleep loss is increasingly recognized as a key public health issue among adolescents. Tobacco use is one of the leading causes of preventable disease and death in the world. Yet, the association between tobacco use and sleep loss has been understudied in the adolescent population. This study aimed to examine this association utilizing nationally representative samples of adolescents.

**Methods:**

Cross-sectional data on 109 408 adolescents (12-15 years) from 38 countries were derived from the Global School-based Student Health Survey (GSHS). Weighted age- and sex-adjusted distribution of each sample characteristics was calculated. Multivariate logistic regression and meta-analyses were performed to assess the association of sleep loss over worry with any tobacco use, while controlling for important confounders, including age, gender, loneliness, physical attack victimization, parental knowledge/warmth, and perceived peer kindness/helpfulness.

**Results:**

The weighted age- and sex-adjusted prevalence of SLOW and use of any tobacco product was 6.4% and 7.4% respectively across 38 countries. The overall odds of sleep loss over worry were 1.89 times (95% confidence interval (CI) = 1.75, 2.03) greater among tobacco users than among non-users, with low level of between-country heterogeneity (*I^2^* = 24.0%, *P* = 0.095). The odds of sleep loss over worry were 1.61 times (95% CI = 1.52, 1.71) greater among those reporting physical attack victimization than among non-victims, and 5.55 times (95% CI = 4.95, 6.21) greater among those reporting frequent than less frequent loneliness.

**Conclusions:**

Tobacco use, physical attack victimization, and loneliness can be key indicators of SLOW, and may be included in the assessment and prevention of SLOW to generate a more comprehensive picture. Further studies are needed to determine if reducing tobacco use, loneliness, or physical attack victimization would make a meaningful impact on reducing SLOW.

Sleep loss is a generic term that broadly describes ‘insufficient sleep’ or ‘less sleep than needed’ [1]. Sleep loss is increasingly viewed as a chronic health problem among adolescents, as it is associated with increased risk of motor vehicle crashes, delinquent behaviors, depression/suicidal ideation, and poor academic performance [1]. Worry has been consistently cited as a cause for sleep loss [2]. Worry refers to future-oriented repetitive thoughts or images about potential threats, uncertainties or risks, and is a core construct in anxiety disorders [3,4]. According to Harvey’s cognitive model of insomnia, excessive negatively toned cognitive activity such as worry is closely implicated in the initiation and continuation of sleep loss [5]. Empirically, worry has been linked to problems with sleep among high trait worriers and patients with generalized anxiety disorder [6,7]. Despite that, worry is a common experience in both adults and adolescents, and it is less extensively studied in relation to sleep in the adolescent population.

Cigarette smoking is considered as a modifiable risk factor for many chronic diseases [8]. Those who initiate cigarette smoking during adolescence are more likely to smoke daily, to continue smoking into adulthood, and to become heavily addicted to nicotine [9]. The positive association between sleep problems (defined broadly) and cigarette smoking has been well-studied in the adult population. A possible mechanism is that nicotine, the highly addictive substance present in cigarettes, exerts its functions through stimulating the release of neurotransmitters (ie, acetylcholine, dopamine) that also help regulate the sleep-wake cycles, contributing to sleep impairment [10]. In addition to cigarettes, nicotine is also present in other tobacco products. Consumption of tobacco products can occur through smoking, chewing, or sniffing. Smoked tobacco products not only include generic cigarettes, but also include cigars (little cigars, cigarillos etc.), bidis (small hand-rolled tobacco-containing cigarettes wrapped in a tendu leaf originated from India), kreteks (clove cigarettes originated from Indonesia), pipes or hookah (water pipe); ground or shredded tobacco that can be chewed or sniffed through the nose are also called smokeless tobacco [11]. Compared with the amount of studies in the adult population, fewer studies examined sleep in relation to tobacco use in the adolescent population. In one such study, Patten et al found cigarette smoking had a dose-response relationship with development of sleep problems among adolescents (12-18 years) in the United States [12]. A meta-analytic review by Kwon et al revealed a positive association between sleep problems and tobacco use (including electronic cigarettes) among adolescents residing in North America [13]. However, these studies were mainly conducted among adolescents in western countries; sleep in relation to tobacco use is less well-studied among adolescents elsewhere. In addition, majority of existing studies focused on the consumption of generic cigarettes, perhaps because they were mostly conducted in western countries where generic cigarettes are the predominant form of tobacco products [14]. Very few studies have taken into consideration the consumption of non-cigarette tobacco products, which are the culturally predominant form of tobacco products in many low- and middle-income countries [14].

In an effort to address these gaps in the literature, data from the Global School-based Student Health Survey (GSHS) was utilized to examine the association of sleep loss over worry (SLOW) - an important indicator of sleep health with any tobacco use among nationally representative samples of adolescents. This study focused on adolescents aged 12-15 exclusively for the following reasons: majority of countries that administered the GSHS sampled students in this age group; smoking is usually initiated during adolescence as most adult smokers have started smoking before the age of 18, and earlier age of smoking initiation is associated with elevated risks of daily smoking and nicotine dependence later in life [10]. Findings of this study may increase the recognition of tobacco use as a key indicator of SLOW among adolescents worldwide, facilitating its inclusion in strategies that help reduce worldwide public health burdens associated with SLOW.

## METHODS

### Data source

Data for this study was derived from the GSHS, which was developed jointly by the World Health Organization, other United Nations-affiliated organizations, and the Center for Disease Control and Prevention in the United States. In each participating country, ethical approval of the survey was obtained from the Ministry of Health or Education as well as an ethics committee. Many items on the GSHS were adopted from the validated Youth Risk Behavior Survey (YRBS) of American adolescents. To ensure data representativeness, the GSHS utilizes a two-stage probability sampling design to recruit participants. At the first stage, schools were selected with probability proportional to the size of student enrollment. Classes were randomly chosen within these schools at the second stage, with all students in selected classes eligible to participate. Informed consent was obtained from students, and from their parents and schools before their participation in the survey. More details about the GSHS are available at https://www.cdc.gov/gshs/pdf/GSHSOVerview.pdf.

### Measures

#### SLOW (outcome variable)

SLOW was the outcome variable, and was assessed by the item: “*During the past 12 months, how often have you been so worried about something that you could not sleep at night?*”. Response options included *1 = “Never”, 2 = “Rarely”, 3 = “Sometimes”, 4 = “Most of the time”,* and *5 = “Always”*. Consistent with other studies utilizing the GSHS data, responses were dichotomized: frequent (*“Most of the time”* and *“Always”*) and infrequent (*“Never”, “Rarely”,* and *“Sometimes”*). This single item was considered a sufficient measure of SLOW for the purpose of this study mainly for three reasons. First, the attribute of the construct being measured is concrete [15]. Second, similar items are embedded in several general health questionnaires (ie, the 12-item General Health Questionnaire) validated for use as screening tools [16]. Third, this study aims to examine the association of SLOW with other constructs, not to make a diagnosis or directly compare individuals.

#### Use of any tobacco product (exposure variable)

Use of any tobacco product was the key exposure variable and assessed by two items: During the past 30 days, “*on how many days did you smoke cigarettes?*” and “*on how many days did you use any tobacco products other than cigarettes*”. Response options included *“0”, “1or 2”, “3 to 5”, “6 to 9”, “10 to 19”, “20 to 29”,* and *“all 30 days”*. The GSHS embedded a dichotomized variable created by combining responses to the two items [17]. The created variable was assigned a value of 1 (*“Yes”*) for those responding 1 day or more to either item, a value of 2 (*“No”*) for those responding 0-day to both items.

#### Confounders

Confounders included age (12, 13, 14, 15 years), gender (male and female), food insecurity (as a proxy for socioeconomic status), feeling of loneliness, victimization by physical attacks, parental knowledge and warmth, and perceived peer kindness and helpfulness. Since the GSHS does not contain items that could directly assess respondents’ socioeconomic status, food insecurity (“*During the past 30 days, how often did you go hungry because there was not enough food in your home?*”) was used as a proxy [18]. Responses were categorized into *“most of the time/always”* vs *“never/rarely/sometimes”*. There is evidence suggesting that sleep duration or quality was affected by age, gender, as well as food insecurity [19,20].

Feeling of loneliness was included as a covariate because lonely individuals across all ages were found to experience worsened sleep quality than non-lonely individuals [21]. Recent evidence suggested a more bidirectional causal relationship between loneliness and sleep loss, as sleep loss may also lead to neural and behavioral changes towards greater loneliness [22]. The GSHS assesses feeling of loneliness using a direct single measure: “*During the past 12 months, how often have you felt lonely?*” Responses were dichotomized into infrequent (*“never”, “rarely”, “sometimes”*), and frequent (*“most of the time”, “always”*) feeling of loneliness. This single-item measure of loneliness is commonly used in population-based surveys worldwide, it may be more easily interpreted by children, and was found to have a significantly positive correlation with multi-item measures such as the UCLA Loneliness Scale [23].

Victimization by physical attacks is a type of physical violence that can lead to sleep problems [24]. It has also been found to be the main source of worry among school-age children [25]. In the GSHS, victimization by physical attacks was assessed by the item “*During the past 12 months, how many times were you physically attacked?*” Response options were dichotomized into “*0 times”* and “*1 or more times”*.

Parental knowledge and warmth are two key dimensions of parenting style. Parental knowledge of their children’s whereabouts or daily activities is more of a function of what their children disclose to them, as it reflects a warm and accepting family environment where the children feel comfortable to disclose information about their lives [26]. Parental warmth is characterized by investing in communication and providing children with the support they need [27]. Some evidence suggests that parental knowledge or warmth was a protective factor against adolescent health risk behaviors [26,27]. In the GSHS, parental knowledge was assessed by “*During the past 30 days, how often did your parents or guardians really know what you were doing with your free time?*” Parental warmth was assessed by: “*During the past 30 days, how often did your parents or guardians understand your problems and worries?*” Responses to these two items were combined to create a dichotomized variable: low (answering *“never”, “rarely”, “sometimes”* to both items) vs high (answering *“most of the time”, “always”* to either item) level of parental knowledge/warmth.

Shared kindness among peers is a key indicator of a positive school climate, which can foster students’ social, emotional well-being and academic achievement, contributing to less violence and aggression [28]. For this reason, it is possible that peer kindness may be a protective factor against SLOW. Perceived peer kindness/helpfulness was assessed in the GSHS by the item: “During the past 30 days, how often were most of the students in your school kind and helpful?” Students responding *“most of the time”* or *“always”* were considered to have high perception of peer kindness/helpfulness vs those responding *“never”, “rarely”,* or *“sometimes”*.

#### Statistical analysis

Countries that lacked data on SLOW, use of any tobacco product, and confounders used in the analysis were excluded. Countries with over 14% of total data missing were further excluded. The final sample consisted of 38 countries in total. The 38 countries were grouped by income level (low-, lower middle-, upper middle-, and high-income) based on the World Bank classification in the year when the survey was conducted in the respective country. Majority of the countries conducted the survey once or twice since 2003. For countries that conducted twice or more, only data from the most recent survey was included. For countries that conducted the same survey within the same year but across multiple cities or areas, data were pooled from all surveyed cities or areas.

Because the GSHS utilized a complex sampling procedure, sampling weights, stratum, and the primary sampling units were included in all statistical analyses. Age- and gender-adjusted distributions of SLOW, use of any tobacco product, and confounders were first estimated for each country. The association of SLOW with use of any tobacco product was estimated for each country via multivariate logistic regression analysis, adjusting for all confounders. All variables were included as categorical variables in the regression analysis with the exception of age (continuous variable). Higgins’s *I*^2^ was calculated to assess the level of between-country heterogeneity. The level of heterogeneity was typically considered low when Higgins’s *I*^2^ was between 25%-50% [29]. A fixed-effect meta-analysis was conducted to obtain the overall estimate of the association of SLOW with tobacco use and confounders when Higgins’s *I^2^* was low, and a random-effect meta analysis was used to obtain estimate of the association when Higgins’s *I^2^* was higher than the recommended value of 25%-50%. Results from logistic regressions were presented as odds ratios (ORs) with 95% confidence intervals (CIs). The level of statistical significance was set at *P* < 0.05. Because Argentina and Malaysia had the largest and second largest sample size, sensitivity analyses were conducted without Argentina or Malaysia or both countries to assess if results were mainly driven by the two countries. All statistical analyses were performed using Stata 14.1 (State Corp LP, College station, Texas, USA).

## RESULTS

The final sample consisted of 109408 adolescents. The overall age- and sex-adjusted prevalence of SLOW and use of any tobacco product was 6.4% (95% CI = 5.2, 7.8; *I^2^* = 93.1%, *P* = 0.000) and 7.4% (95% CI = 6.2, 8.9; *I^2^* = 86.1%, *P* = 0.000) respectively, with significant between-country heterogeneity. At the country level, the age- and sex-adjusted prevalence of SLOW ranged from 0.5% in Myanmar to 15.7% in West Bank and Gaza, while use of any tobacco product ranged from 2.0% in Myanmar to 25.1% in Kiribati (**Table 1**). Of all 38 countries, 34 countries have ratified the WHO Framework Convention on Tobacco Control (FCTC) (**Table 2**). Argentina and Morocco have signed but not ratified the WHO FCTC; Indonesia, West Bank and Gaza are neither signatories nor Parties to the WHO FCTC.

**Table 1:** Survey characteristics by country (N = 109 408). GSHS, 2005-2015

Income level*	Country	WHO	Survey	Response	N (Total)‡	Male (%)§	Food insecurity
**Region**	**Year**	**rate (%)†**	**% (95%CI)§‖**
LIC	Bangladesh	SEAR	2014	91%	2472	63.3	9.0 (5.0, 13.0)
	Benin	AFR	2009	90%	1138	66.4	9.3 (5.9, 12.8)
	Myanmar	SEAR	2007	95%	2142	48.9	2.6 (1.1, 4.2)
LMIC	Bolivia	AMR	2012	88%	2607	49.8	9.6 (6.9, 12.3)
	Djibouti	EMR	2007	83%	879	58.6	9.0 (5.4, 12.6)
	Guyana	AMR	2010	76%	1835	48.2	4.7 (3.3, 6.0)
	Honduras	AMR	2012	79%	1373	45.4	3.8 (2.2, 5.4)
	Indonesia	SEAR	2015	94%	8410	48.7	3.3 (2.5, 4.0)
	Kiribati	SEAR	2011	85%	1237	44.5	12.0 (8.0, 16.1)
	Mauritania	AFR	2010	70%	1099	52.4	4.8 (2.1, 7.5)
	Mongolia	WPR	2013	88%	3584	49.0	1.0 (0.5, 1.5)
	Morocco	EMR	2010	92%	2149	52.5	6.8 (5.0, 8.5)
	Pakistan	EMR	2009	76%	4698	60.3	4.3 (2.6, 6.1)
	Philippines	WPR	2011	82%	3636	47.7	6.0 (3.7, 8.3)
	Solomon Islands	WPR	2011	85%	802	50.8	6.9 (3.2, 10.6)
	Thailand	SEAR	2008	89%	2478	46.3	3.4 (2.3, 4.5)
	Tonga	WPR	2010	80%	1742	49.9	11.7 (8.6, 14.8)
	West Bank and Gaza¶	EMR	2010	94%, 95%	3758	48.0	10.6 (8.6, 12.5)
UMIC	Argentina	AMR	2012	71%	19083	47.4	2.3 (1.6, 3.1)
	Botswana	AFR	2005	95%	1223	45.9	8.4 (2.1, 14.7)
	Cook Islands	WPR	2015	65%	352	48.2	2.6 (0.4, 4.8)
	Iraq	EMR	2012	88%	1400	54.4	7.2 (3.8, 10.7)
	Jamaica	AMR	2010	72%	1063	49.6	7.1 (4.5, 9.7)
	Jordan	EMR	2007	99.8%	1431	45.2	10.9 (6.0, 15.9)
	Malaysia	WPR	2012	89%	15844	49.2	2.9 (1.8, 3.9)
	Maldives	SEAR	2009	80%	1749	47.4	2.5 (0.5, 4.5)
	Namibia	AFR	2013	89%	1786	42.2	3.2 (1.7, 4.8)
	Peru	AMR	2010	85%	2288	49.8	3.6 (0.5, 6.7)
	St Lucia	AMR	2007	82%	955	43.9	5.6 (3.2, 8.0)
	Suriname	AMR	2009	89%	976	44.7	4.3 (2.8, 5.7)
	Tunisia	EMR	2008	83%	2286	47.4	6.3 (4.8, 7.9)
HIC	The Bahamas	AMR	2013	78%	1136	46.5	6.1 (3.1, 9.1)
	Barbados	AMR	2011	73%	1350	50.6	2.5 (1.0, 4.1)
	Brunei Darussalam	SEAR	2014	65%	1722	48.1	4.0 (2.5, 5.5)
	Kuwait	EMR	2015	78%	1786	50.1	2.2 (0.6, 3.8)
	Trinidad and Tobago	AMR	2011	90%	2155	48.9	4.2 (3.2, 5.2)
	United Arab Emirates	EMR	2010	91%	2126	38.3	3.8 (2.0, 5.7)
	Uruguay	AMR	2012	77%	2658	45.6	1.1 (0.6, 1.7)

HIC – high income countries, UMIC – upper middle income countries, LIC – low income countries, LMIC – lower middle income countries, AFR – African region, AMR – Region of the Americas, EMR – Eastern Mediterranean Region, SEAR – Southeast Asia Region, WPR – Western Pacific Region

*Country income level was based on the World Bank classification at the year of the survey in the respective countries.

†Response rate = school response rate × student response rate.

‡Based on sample students aged 12-15 years.

§Estimates were weighted.

‖Were hungry most of the time or always because there was not enough food in their home in the past 30 days. Estimates were sex- and age-adjusted.

¶Occupied Palestinian territory.

**Table 2:** Age- and sex-adjusted prevalence of past 12-mo sleep loss over worry, and past 30-d any tobacco use among adolescents aged 12-15 (N = 109 408), and year WHO FCTC was ratified for each country. GSHS, 2005-2015

Income level*	Country	Sleep loss over worry	Use of any tobacco product	Year WHO FCTC ratified
**% (95% CI) †‡**	**% (95% CI)†§**
LIC	Bangladesh	6.6 (1.0, 12.2)	7.3 (0.6, 14.0)	2004
	Benin	7.9 (5.3, 10.5)	2.5 (1.6, 3.5)	2005
	Myanmar	0.5 (0.1, 1.0)	2.0 (0.1, 4.0)	2004
LMIC	Bolivia	5.4 (3.8, 7.0)	8.6 (6.1, 11.0)	2005
	Djibouti	4.6 (2.0, 7.2)	3.6 (1.2, 6.0)	2005
	Guyana	14.1 (6.3, 21.9)	15.6 (4.6, 26.6)	2005
	Honduras	3.9 (1.7, 6.2)	8.5 (5.1, 12.0)	2005
	Indonesia	3.1 (2.4, 3.8)	7.1 (5.4, 8.9)	not ratified
	Kiribati	6.0 (2.6, 9.5)	25.1 (15.9, 34.2)	2005
	Mauritania	6.7 (2.8, 10.6)	12.5 (7.4, 17.7)	2005
	Mongolia	3.2 (2.4, 4.1)	3.9 (2.8, 5.0)	2004
	Morocco	10.3 (7.8, 12.8)	4.4 (2.8, 6.1)	not ratified
	Pakistan	5.1 (3.0, 7.3)	8.1 (5.5, 10.7)	2004
	Philippines	7.8 (4.9, 10.6)	7.2 (4.4, 10.0)	2005
	Solomon Islands	9.5 (5.0, 14.1)	10.6 (4.6, 16.5)	2004
	Thailand	5.3 (3.8, 6.7)	6.1 (3.8, 8.3)	2004
	Tonga	10.0 (7.1, 12.9)	17.6 (12.6, 22.6)	2005
	West Bank and Gaza	15.7 (12.3, 19.2)	20.6 (15.3, 26.0)	not ratified
UMIC	Argentina	5.3 (4.2, 6.5)	8.9 (7.4, 10.3)	not ratified
	Botswana	12.2 (5.8, 18.7)	4.2 (1.1, 7.2)	2005
	Cook Islands	6.5 (2.7, 10.2)	6.9 (1.0, 12.7)	2004
	Iraq	7.1 (5.9, 8.4)	6.7 (4.2, 9.2)	2008
	Jamaica	13.4 (8.4, 18.4)	21.5 (11.6, 31.4)	2005
	Jordan	14.6 (9.5, 19.8)	13.3 (8.1, 18.5)	2004
	Malaysia	1.8 (1.4, 2.2)	5.9 (3.8, 8.0)	2005
	Maldives	7.0 (2.3, 11.7)	4.6 (1.4, 7.7)	2004
	Namibia	4.7 (2.6, 6.9)	3.5 (0.8, 6.2)	2005
	Peru	5.7 (3.4, 8.0)	10.3 (6.7, 13.9)	2004
	St Lucia	8.2 (6.0, 10.4)	8.6 (5.1, 12.1)	2005
	Suriname	3.3 (1.7, 4.9)	3.8 (2.3, 5.3)	2008
	Tunisia	14.2 (12.1, 16.3)	5.8 (4.4, 7.3)	2010
HIC	The Bahamas	10.4 (7.3, 13.6)	6.2 (3.9, 8.5)	2009
	Barbados	6.3 (4.6, 8.0)	7.0 (4.7, 9.3)	2005
	Brunei Darussalam	4.3 (3.0, 5.6)	4.2 (2.5, 5.9)	2004
	Kuwait	8.1 (6.2, 10.0)	7.7 (3.3, 12.0)	2006
	Trinidad and Tobago	3.1 (2.5, 3.8)	3.9 (2.2, 5.6)	2004
	United Arab Emirates	9.0 (6.8, 11.2)	7.2 (4.2, 10.1)	2005
	Uruguay	4.9 (0.1, 9.8)	6.4 (1.4, 11.4)	2004

WHO FCTC – WHO Framework Convention on Tobacco Control, LIC – low income countries, LMIC – lower middle income countries, UMIC – upper middle income countries, HIC – high income countries, CI – confidence interval

*Country income level was based on the World Bank classification at the year of the survey in the respective countries.

†Estimates are weighted, sex- and age-adjusted.

‡Were worried about something that they could not sleep at night most of the time or always in the past 12 months.

§Used any tobacco products on one or more days in the past 30 days.

Country-specific age- and sex-adjusted prevalence of each confounder is summarized in Table S1 in **Online Supplementary Document**. The overall age- and sex-adjusted prevalence of victimization by physical attacks was 26.7%, with significant between-country heterogeneity (*I^2^* = 93.9%, *P* = 0.000) ranging from 9.9% in Kiribati to 56.5% in Bangladesh. The overall age- and sex-adjusted prevalence of loneliness was 7.6%, with significant between-country heterogeneity (*I^2^* = 87.0%, *P* = 0.000) ranging from 2.7% in Myanmar and Uruguay to 13.3% in Tunisia. The overall age- and sex-adjusted prevalence of parental knowledge/warmth was 39.7%, with significant between-country heterogeneity (*I^2^* = 97.1%, *P* = 0.000) ranging from 15.7% in West Bank and Gaza to 68.9% in Uruguay. And the overall age- and sex-adjusted prevalence of perceived peer kindness/helpfulness was 27.3%, with significant between-country heterogeneity (*I^2^* = 95.5%, *P* = 0.000) ranging from 10.7% in Namibia to 61.5% in Uruguay.

**Figure 1:**
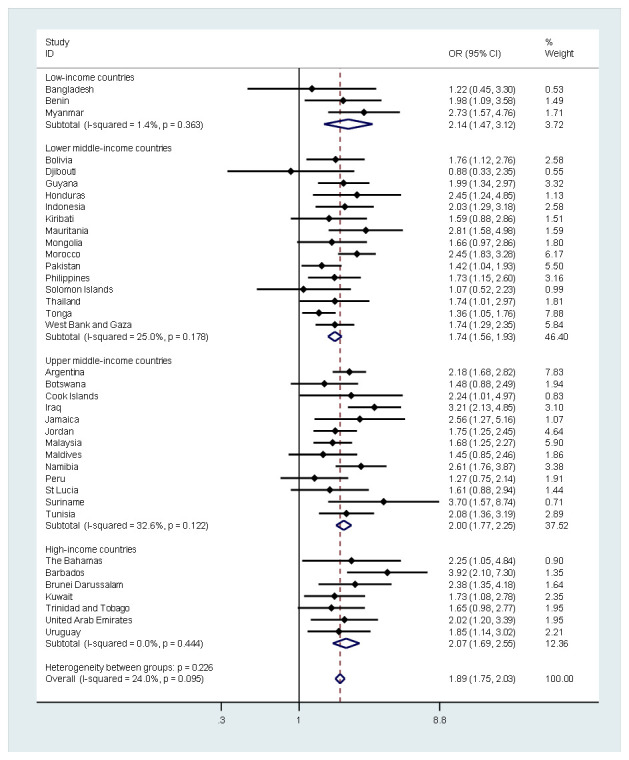
Country-wise association between past 30-day use of any tobacco product (1 or more days) and past 12-month frequency of sleep loss over worry (most of the time/always) estimated by multivariate logistic regression. OR – odds ratio, CI – confidence interval. Models were adjusted for age, sex, food insecurity, loneliness, parental monitoring, kind/helpful peers, and physical attack victimization. Overall estimates were obtained by meta-analysis with fixed effects.

The association of SLOW with use of any tobacco product by country and country-income level is presented in **Figure 1**. Overall, the odds of SLOW among tobacco users were nearly twice (AOR = 1.89; 95% CI = 1.75, 2.03) as high as among non-users, while the level of between-country heterogeneity was low (*I^2^* = 24.0%, *P* = 0.095). At every country-income level, SLOW was also significantly associated with use of any tobacco product, with odds ratios ranging from 1.74 (95% CI = 1.56, 1.93) in lower middle-income countries to 2.14 (95% CI = 1.47, 3.12) in low-income countries. Sensitivity analysis showed that these results were similar with or without Argentina and Malaysia, suggesting that the results were not mainly driven by countries with larger sample sizes.

Country-wise association of SLOW with each confounder is summarized in Table S2 in **Online Supplementary Document**. Overall, significant between-country heterogeneity was observed in the association of SLOW with each confounder except for physical attack victimization. Compared with females, male respondents had 37% (95% CI = 0.56, 0.72) reduced odds of reporting SLOW. The odds of reporting SLOW among respondents with frequent food insecurity were nearly twice as high as (AOR = 1.87; 95% CI = 1.67, 2.09) among those with less frequent food insecurity. The odds of SLOW were highest among respondents experiencing frequent loneliness (AOR = 5.55; 95% CI = 4.95, 6.21). Furthermore, the odds of SLOW were 1.61 (95% CI = 1.52, 1.71) times greater among victims of physical attacks than among non-victims. However, SLOW was not significantly associated with parental knowledge/warmth or perceived peer kindness/helpfulness across all countries.

## DISCUSSION

This is the first cross-national study that focused explicitly on the association of SLOW with use of any tobacco product among large representative samples of 12- to 15-year-old adolescents. The main finding was that frequent SLOW among tobacco users was nearly twice (AOR = 1.89; 95% CI = 1.76, 2.03) as high as among non-users, while the level of between-country heterogeneity (*I^2^* = 24.0%, *P* = 0.095) was low. Moreover, in majority of the 38 countries, statistical significance of the association between SLOW and any tobacco use was evident over and above the variance accounted for by age, gender, food insecurity, loneliness, victimization by physical attacks, parental knowledge/warmth, and perceived peer kindness/helpfulness.

Previous studies consistently documented the link between cigarette smoking and difficulties getting to sleep and difficulties staying asleep [30], attributing the connection largely to the effects of nicotine. Nicotine is an addictive substance present in all tobacco products, it can stimulate the release of neurotransmitters such as acetylcholine, dopamine, serotonin, and glutamate, which are also involved in the regulation of the sleep-wake cycle [31]. For example, when acetylcholine binds to its receptors, it mainly causes cognitive arousal; while nicotine mimics the effect of acetylcholine and binds to nicotinic acetylcholinergic receptors, causing the release of excitatory neurotransmitters, which in turn cause cognitive arousal and interfere with sleep onset and staying asleep [31]. The current study corroborated the positive association of disturbed sleep with tobacco use in the adolescent population across 38 countries, suggesting a more universal significance of this association, though its temporal sequence still needs to be determined through longitudinal studies. Yet, smoking has served as a coping strategy to reduce worrying among those with high trait anxiety [32], further research is needed to parse out the unique variance in sleep loss explained by worry and by tobacco use respectively.

Findings of this study also showed that, approximately 7 or 8 out of every 100 (7.6%) responding adolescents felt lonely most of the time or always during the past 12 months. Although loneliness is thought to be more prevalent in the elderly population, yet, findings from recent studies suggest that loneliness might best characterize the younger rather than the older population [33]. The association of sleep with loneliness has been less often studied than its association with mental health in the adolescent population. One proposed mechanism through which loneliness can affect sleep is that, loneliness can trigger a state of hypervigilance for social threat and augment anxiety or depression [34]. In the current study, the association of SLOW with loneliness was robust across all countries, and the overall odds of SLOW among respondents experiencing frequent loneliness were more than 5 times (AOR = 5.55; 95% CI = 4.95, 6.21) as high as those experiencing less frequent loneliness. With country-specific odds ratios ranging from 2.21 to 15.6, the level of between-country heterogeneity (*I^2^* = 67.6%, *P* = 0.000) further suggested that the association was more pronounced in some countries, though the underlying causes of such a difference remain to be determined. Nonetheless, this finding underscores the significance of including loneliness reduction in adolescent SLOW prevention, especially in countries where the association was most evident.

Experiencing physical attacks has been cited as a main source of worry among children and adolescents [25], it can incur long-lasting negative physical and mental health conditions. In the adolescent population, victimization by physical attacks is often examined as a form of overt peer victimization. However, physical attacks can occur outside of the school environment, and perpetrators can be anyone besides peers. Although several studies linked physical victimization to bedtime fears and to more sleep disturbance [35], its association with sleep has been sparsely researched. This study found slightly more than a quarter (26.7%) of respondents fell victims to physical attacks during the past year, and the odds of SLOW among victims of physical attacks were 1.61 times (95% CI = 1.52, 1.71) as high as those not victimized. The low level of between-country heterogeneity in this association further corroborated the necessity to address reducing physical violence in improving sleep health among adolescents from countries where their association was especially pronounced.

Even though parental knowledge/warmth was hypothesized to be an essential protective factor against SLOW, this study did not find its association with SLOW statistically significant overall. However, substantial between-country heterogeneity in the magnitude as well as direction of the association was observed, suggesting that country-specific factors may have contributed to the interpretation of parental knowledge/warmth and to its varying association with SLOW. Similarly, SLOW was not significantly associated with perceived peer kindness/helpfulness in this study. Although perceived peer kindness/helpfulness is a key aspect of a positive school climate [28], there is limited evidence supporting its link with sleep health. One study found a positive link between sleep quality and perceived school climate, yet, perceived peer kindness/helpfulness and its link to sleep quality was not separately assessed and could not be directly determined [36]. Further research is needed to examine the exact roles of parental knowledge/warmth and peer kindness/helpfulness in determining sleep health.

### Limitations

The strengths of the study included nationally representative samples of adolescents from multiple low- and middle-income countries, adjustment for key confounders such as loneliness and physical attacks, which have not been fully adjusted for in previous studies. Yet, this study also has several limitations. First, data on variables used in the analysis were mainly derived from responses to a single self-report item, which may fall short of capturing the full breadth of a construct. Although it is common to use single-item measures in population-based surveys, caution should be taken when comparing the results against those obtained through using multidimensional instruments. However, in addition to reducing common method variance [37], single-item measures may be more easily interpreted by children and more cost-effective to administer in population-based surveys where survey space is a key constraint. Second, due to the cross-sectional design of the survey, the temporal sequence of SLOW and tobacco use cannot be fully derived, more longitudinal studies are needed to establish causality of this association in different adolescent populations.

## CONCLUSION

Since insufficient sleep is linked to many health problems including obesity, diabetes, poor mental health, and injuries [1], and frequent worry-related insufficient sleep may reflect underlying issues pertaining to self (trait worry) and/or peripheral influencers (lifestyle and environmental factors) [38], identifying key factors strongly linked to insufficient sleep may facilitate the recognition of individuals at risk of developing sleep-related problems. The association between tobacco use and sleep disturbance was well-documented in both clinical and healthy adult populations, but was underexplored in the adolescent population. The current study revealed the negative role of tobacco use in sleep health as SLOW was robustly associated with tobacco use among adolescents aged 12-15 in majority of the countries, even after controlling for significant confounders. Furthermore, the current study drew attention to the unique roles of physical attack victimization and loneliness in SLOW, which were also underexplored in previous studies. Together, these findings implied that tobacco use, physical attack victimization, and loneliness can be key indicators of SLOW, and need to be included in the assessment and prevention of SLOW to generate a more comprehensive picture. Nonetheless, further studies are needed to determine if reducing tobacco use, frequent loneliness, or physical attack victimization would make a meaningful impact on reducing SLOW.

## Additional material

Online Supplementary Document
