# Supplementary material for: Tobacco use and sleep loss over worry among adolescents aged 12-15 years: A population-based study of 38 countries
Source: J Glob Health. 2020 Dec 6;10(2):020427. doi: 10.7189/jogh.10.020427 (PMC7719269; doi:10.7189/jogh.10.020427)
Supplement: Online Supplementary Document [file jogh-10-020427-s001.pdf]

Supplementary Table 1. Country-wise age- and sex-adjusted prevalence of reporting physical attacks, feeling lonely, parental monitoring, and kind/helpful peers (N=109,408). GSHS, 2005-2015.

Income level[a]	Country	Victimization by physical attacks % (95%CI)[b,e]	Feeling of loneliness % (95%CI)[b,f]	Parental knowledge/warmth % (95%CI)[b,g]	Perceived peer kindness/helpfulness % (95%CI)[b,h]
**LIC**	Bangladesh	56.5 (43.0, 70.0)	10.0 (3.4, 16.7)	68.8 (63.2, 74.3)	51.3 (44.2, 58.5)
	Benin	18.7 (15.8, 21.6)	7.0 (4.0, 10.1)	33.8 (30.7, 36.8)	21.5 (18.6, 24.4)
	Myanmar	25.9 (17.1, 34.8)	2.7 (1.4, 4.0)	19.6 (14.2, 25.1)	23.8 (17.8, 30.0)
**LMIC**	Bolivia	27.4 (24.3, 30.4)	6.7 (4.6, 8.8)	38.9 (35.7, 42.1)	29.3 (25.4, 33.3)
	Djibouti	31.0 (26.4, 35.5)	7.9 (4.4, 11.3)	29.1 (24.3, 34.0)	11.6 (7.6, 15.5)
	Guyana	36.1 (25.0, 47.1)	12.4 (4.9, 19.8)	50.3 (39.5, 61.1)	26.1 (20.3, 32.0)
	Honduras	19.0 (15.1, 22.9)	8.3 (6.0, 10.5)	58.0 (54.5, 61.5)	34.7 (31.9, 37.6)
	Indonesia	29.3 (27.0, 31.7)	3.8 (3.0, 4.6)	41.2 (38.9, 43.5)	29.2 (26.9, 31.6)
	Kiribati	9.9 (4.2, 15.5)	4.8 (1.9, 7.6)	23.3 (18.2, 28.5)	20.2 (13.7, 26.6)
	Mauritania	34.2 (27.7, 40.7)	10.6 (6.3, 15.0)	31.5 (26.7, 36.2)	23.2 (17.6, 28.6)
	Mongolia	21.2 (19.3, 23.1)	8.6 (7.1, 10.2)	41.3 (39.5, 43.2)	17.5 (15.7, 19.3)
	Morocco	19.8 (15.7, 23.8)	11.4 (8.2, 14.5)	44.4 (40.3, 48.4)	28.3 (24.1, 32.6)
	Pakistan	31.1 (23.4, 38.7)	7.5 (5.5, 9.5)	64.3 (58.2, 70.4)	38.4 (33.7, 43.0)
	Philippines	24.3 (18.4, 30.2)	9.3 (7.2, 11.5)	33.1 (26.5, 39.7)	22.3 (16.6, 27.9)
	Solomon Islands	34.1 (26.7, 41.5)	8.6 (5.2, 12.0)	30.8 (24.8, 36.9)	20.7 (16.1, 25.2)
	Thailand	36.1 (29.9, 42.3)	5.6 (4.2, 7.1)	40.2 (35.8, 44.7)	23.5 (18.6, 28.5)
	Tonga	39.5 (34.0, 45.1)	12.2 (9.1, 15.3)	36.2 (30.5, 42.0)	32.5 (26.4, 38.5)
	West Bank and Gaza	47.3 (43.5, 51.0)	12.4 (10.3, 14.5)	15.7 (12.3, 19.2)	20.6 (15.3, 26.0)
**UMIC**	Argentina	21.9 (19.2, 24.7)	5.8 (4.8, 6.7)	57.6 (54.4, 60.8)	36.7 (34.0, 39.4)

	Botswana	33.7 (27.8, 39.6)	8.0 (4.9, 11.0)	29.4 (22.9, 35.9)	19.9 (12.6, 27.2)
	Cook Islands	20.7 (12.7, 28.6)	4.6 (1.9, 7.4)	23.5 (13.4, 33.6)	21.7 (11.5, 31.8)
	Iraq	25.5 (21.7, 29.4)	10.2 (8.0, 12.3)	46.3 (39.3, 53.4)	32.8 (26.0, 39.6)
	Jamaica	28.3 (18.9, 37.7)	10.6 (3.7, 17.6)	36.4 (23.9, 48.9)	19.8 (11.5, 28.1)
	Jordan	29.3 (23.2, 35.5)	11.7 (6.9, 16.4)	40.9 (34.6, 47.2)	25.7 (19.6, 31.8)
	Malaysia	20.7 (17.8, 23.5)	3.4 (2.3, 4.5)	36.4 (33.6, 39.1)	24.9 (22.1, 27.7)
	Maldives	22.0 (17.8, 26.3)	9.0 (6.5, 11.4)	35.7 (29.7, 41.6)	33.5 (29.4, 37.6)
	Namibia	16.5 (12.5, 20.6)	3.7 (2.6, 4.8)	22.6 (20.3, 24.8)	10.7 (6.9, 14.5)
	Peru	32.8 (26.9, 38.8)	7.1 (4.2, 10.0)	45.6 (40.3, 50.9)	36.7 (30.2, 43.1)
	St Lucia	32.0 (26.4, 37.7)	10.2 (7.8, 12.6)	34.4 (31.3, 37.5)	28.8 (25.4, 32.2)
	Suriname	15.6 (12.5, 18.8)	8.3 (5.1, 11.5)	41.3 (37.5, 45.1)	31.5 (27.7, 35.3)
	Tunisia	41.9 (39.2, 44.7)	13.3 (11.2, 15.4)	40.2 (37.3, 43.1)	34.2 (31.3, 37.0)
**HIC**	The Bahamas	24.5 (20.6, 28.4)	9.0 (6.5, 11.6)	62.5 (58.4, 66.7)	26.9 (20.3, 33.5)
	Barbados	23.7 (19.2, 28.2)	10.5 (7.3, 13.8)	54.1 (48.6, 59.6)	15.9 (11.5, 20.2)
	Brunei Darussalam	23.8 (20.3, 27.2)	7.2 (5.0, 9.3)	40.0 (36.5, 43.4)	38.1 (34.4, 41.9)
	Kuwait	19.7 (16.5, 23.0)	8.1 (7.0, 9.3)	34.0 (31.3, 36.7)	25.4 (23.2, 27.5)
	Trinidad and Tobago	25.0 (21.4, 28.6)	5.8 (4.9, 6.7)	57.9 (54.7, 61.2)	25.7 (21.6, 29.9)
	United Arab Emirates	31.5 (24.8, 38.1)	11.3 (7.0, 15.7)	61.1 (56.3, 65.9)	55.9 (50.7, 61.2)
	Uruguay	16.6 (5.9, 27.2)	2.7 (1.6, 3.9)	68.9 (63.0, 74.7)	61.5 (55.8, 67.1)

[a]Country income level was based on the World Bank classification at the year of the survey in the respective countries. LIC=low income countries; LMIC=lower middle income countries; UMIC=upper middle income countries; HIC=high income countries.

[b]Estimates were weighted, sex- and age-adjusted.

[c]Were worried about something that they could not sleep at night most of the time or always in the past 12 months.

[d]Used any tobacco products on at least one day in the past 30 days.

[e]Were physically attacked at least one time in the past 12 months.

[f]Felt lonely most of the time or always in the past 12 months.

[g]Parents understood their problems and worries, or know what they were doing with their free time most of the time or always in the past 30 days.

[h]Most of the students in their school were kind and helpful most of the time or always in the past 30 days.

Supplementary Table 2. Country-wise multivariate logistic regression results for adolescents aged 12-15 (N=109,408). GSHS, 2005-2015.

Country	Age	Gender	Food insecurity	Feeling of loneliness[a]	Victimization by physical attacks[b]	Parental knowledge/warmth[c]	Perceived peer kindness/helpfulness[d]
	AOR (95% CI)	AOR (95% CI)	AOR (95% CI)	AOR (95% CI)	AOR (95% CI)	AOR (95% CI)	AOR (95% CI)
**LICs**							
Bangladesh	0.99 (0.68, 1.42)	0.71 (0.34, 1.50)	1.28 (0.68, 2.42)	7.43 (3.99, 13.83)***	1.10 (0.60, 2.01)	0.73 (0.38, 1.43)	0.88 (0.44, 1.76)
Benin	1.12 (0.98, 1.28)	1.24 (0.90, 1.72)	2.00 (0.90, 4.45)	4.04 (2.68, 6.09)***	1.55 (1.17, 2.07)**	1.30 (0.91, 1.87)	1.04 (0.80, 1.36)
Myanmar	1.55 (1.09, 2.19)*	1.03 (0.47, 2.24)	3.87 (0.36, 41.5)	15.6 (7.50, 32.5)***	1.94 (0.81, 4.68)	1.11 (0.38, 3.23)	0.78 (0.26, 2.38)
**LMICs**							
Bolivia	0.96 (0.82, 1.12)	0.62 (0.43, 0.91)*	2.15 (1.17, 3.94)*	9.90 (7.11, 13.79)***	1.95 (1.37, 2.78)***	0.88 (0.61, 1.28)	0.95 (0.58, 1.53)
Djibouti	1.32 (1.04, 1.68)*	0.72 (0.46, 1.13)	1.87 (1.07, 3.26)*	2.70 (1.63, 4.46)***	1.80 (1.10, 2.94)*	1.41 (0.86, 2.31)	1.08 (0.63, 1.88)
Guyana	0.90 (0.75, 1.09)	0.88 (0.58, 1.35)	0.93 (0.52, 1.64)	4.71 (3.25, 6.82)***	1.49 (1.09, 2.02)*	0.87 (0.57, 1.34)	1.19 (0.83, 1.72)
Honduras	1.21 (0.87, 1.68)	1.04 (0.58, 1.84)	1.56 (0.57, 4.23)	7.00 (2.76, 17.78)***	2.85 (1.67, 4.85)***	1.42 (0.68, 2.98)	1.44 (0.62, 3.32)
Indonesia	1.07 (0.94, 1.20)	0.94 (0.68, 1.29)	1.79 (1.20, 2.66)**	8.09 (5.46, 12.00)***	1.64 (1.25, 2.15)***	1.01 (0.76, 1.35)	1.15 (0.91, 1.45)
Kiribati	1.15 (0.87, 1.53)	1.00 (0.69, 1.44)	1.40 (0.76, 2.58)	3.68 (1.88, 7.18)***	2.64 (1.60, 4.36)***	1.75 (1.09, 2.82)*	1.19 (0.65, 2.20)
Mauritania	0.97 (0.70, 1.34)	0.88 (0.51, 1.51)	1.65 (1.04, 2.61)*	7.20 (4.22, 12.29)***	1.29 (0.78, 2.14)	1.42 (0.78, 2.58)	0.59 (0.39, 0.88)**
Mongolia	1.13 (0.96, 1.33)	1.20 (0.88, 1.64)	3.93 (1.57, 9.85)**	9.39 (6.78, 13.00)***	1.42 (0.93, 2.17)	0.60 (0.42, 0.86)**	1.12 (0.77, 1.62)
Morocco	1.03 (0.91, 1.17)	0.54 (0.38, 0.77)**	1.85 (1.27, 2.71)**	6.45 (4.49, 9.22)***	1.50 (1.15, 1.95)**	1.04 (0.80, 1.34)	1.01 (0.75, 1.36)
Pakistan	1.21 (1.06, 1.38)**	0.82 (0.57, 1.17)	2.64 (1.73, 4.02)***	7.84 (5.82, 10.56)***	1.39 (1.02, 1.89)*	0.90 (0.64, 1.25)	1.06 (0.80, 1.42)
Philippines	1.01 (0.84, 1.20)	0.78 (0.58, 1.03)	1.88 (1.05, 3.36)*	4.18 (3.16, 5.52)***	1.49 (1.20, 1.84)***	1.10 (0.82, 1.49)	1.38 (1.09, 1.75)**
Solomon Islands	0.93 (0.66, 1.29)	0.85 (0.50, 1.43)	1.81 (0.90, 3.62)	3.16 (1.43, 6.96)**	2.28 (1.26, 4.15)**	1.06 (0.52, 2.15)	0.75 (0.45, 1.24)
Thailand	1.00 (0.86, 1.17)	0.64 (0.41, 1.01)	2.69 (1.43, 5.06)**	9.68 (5.96, 15.73)***	2.00 (1.18, 3.38)*	1.45 (1.09, 1.92)*	0.84 (0.54, 1.29)
Tonga	1.25 (1.02, 1.53)*	0.82 (0.61, 1.08)	1.74 (1.12, 2.71)*	5.25 (3.96, 6.95)***	1.03 (0.74, 1.42)	1.08 (0.80, 1.46)	0.95 (0.70, 1.29)
West Bank and Gaza[e]	1.07 (0.96, 1.20)	0.45 (0.36, 0.55)***	1.95 (1.50, 2.54)***	4.28 (3.34, 5.49)***	1.51 (1.18, 1.92)**	0.87 (0.68, 1.10)	1.04 (0.84, 1.28)
**UMICs**							
Argentina	1.05 (0.91, 1.21)	0.44 (0.36, 0.54)***	1.08 (0.67, 1.76)	6.37 (4.85, 8.38)***	2.41 (1.75, 3.31)***	0.70 (0.55, 0.88)**	1.09 (0.88, 1.35)
Botswana	1.14 (0.87, 1.49)	0.95 (0.72, 1.27)	1.80 (1.10, 2.96)*	2.21 (1.40, 3.47)**	1.60 (1.18, 2.16)**	1.37 (1.05, 1.78)*	1.22 (0.82, 1.81)
Cook Islands	1.29 (0.63, 2.65)	0.50 (0.24, 1.04)	0.51 (0.08, 3.24)	5.28 (2.07, 13.48)**	1.35 (0.72, 2.55)	0.84 (0.45, 1.55)	1.26 (0.52, 3.06)
Iraq	1.11 (0.93, 1.31)	0.34 (0.22, 0.54)***	2.74 (1.42, 5.29)**	8.10 (4.90, 13.37)***	1.20 (0.72, 2.02)	0.83 (0.54, 1.25)	0.81 (0.53, 1.22)
Jamaica	0.87 (0.71, 1.07)	0.47 (0.27, 0.80)**	1.72 (1.38, 2.14)***	4.71 (3.14, 7.08)***	1.13 (0.77, 1.66)	0.67 (0.44, 1.03)	1.88 (1.38, 2.57)***

Jordan	1.00 (0.83, 1.21)	0.38 (0.26, 0.56)***	1.71 (1.07, 2.73)*	5.45 (3.39, 8.78)***	1.24 (0.80, 1.92)	1.11 (0.81, 1.52)	1.25 (0.94, 1.66)
Malaysia	1.12 (1.01, 1.25)*	0.77 (0.60, 0.98)*	1.74 (1.17, 2.59)**	7.38 (5.56, 9.80)***	2.04 (1.62, 2.56)***	0.97 (0.78, 1.19)	1.01 (0.84, 1.21)
Maldives	1.38 (1.11, 1.72)**	0.48 (0.29, 0.77)**	3.10 (1.69, 5.70)***	6.06 (3.94, 9.30)***	1.63 (1.09, 2.46)*	0.64 (0.44, 0.93)*	1.12 (0.73, 1.72)
Namibia	1.16 (0.99, 1.36)	0.83 (0.59, 1.16)	3.35 (2.34, 4.79)***	3.27 (2.33, 4.60)***	1.64 (1.13, 2.39)*	1.02 (0.80, 1.29)	1.28 (0.90, 1.82)
Peru	0.96 (0.82, 1.11)	0.61 (0.45, 0.82)**	1.81 (0.93, 3.50)	5.42 (3.89, 7.55)***	1.40 (0.89, 2.20)	1.09 (0.78, 1.51)	0.65 (0.44, 0.95)*
St Lucia	1.11 (0.88, 1.41)	0.54 (0.29, 1.02)	3.03 (1.34, 6.83)**	5.13 (3.30, 7.98)***	1.15 (0.72, 1.85)	1.03 (0.67, 1.60)	1.08 (0.62, 1.86)
Suriname	1.39 (1.08, 1.79)*	0.65 (0.25, 1.70)	1.16 (0.58, 2.34)	6.42 (3.33, 12.38)***	2.08 (1.42, 3.04)**	0.91 (0.46, 1.78)	0.81 (0.52, 1.27)
Tunisia	1.09 (0.92, 1.28)	0.42 (0.30, 0.59)***	1.74 (1.15, 2.65)*	6.18 (5.34, 7.16)***	1.74 (1.23, 2.47)**	1.10 (0.82, 1.47)	1.12 (0.80, 1.56)
**HICs**							
The Bahamas	1.21 (0.93, 1.58)	0.50 (0.34, 0.74)**	2.37 (1.19, 4.71)*	3.07 (1.99, 4.74)***	1.67 (1.12, 2.49)*	0.63 (0.40, 0.98)*	1.08 (0.64, 1.82)
Barbados	0.93 (0.73, 1.19)	0.35 (0.22, 0.56)***	2.27 (1.09, 4.73)*	5.23 (3.03, 9.03)***	1.56 (1.02, 2.38)*	0.76 (0.49, 1.19)	0.53 (0.25, 1.09)
Brunei Darussalam	1.25 (1.00, 1.55)*	0.48 (0.32, 0.71)***	1.30 (0.71, 2.38)	4.67 (2.86, 7.64)***	2.11 (1.47, 3.04)***	0.85 (0.58, 1.23)	1.28 (0.75, 2.17)
Kuwait	1.02 (0.85, 1.23)	0.43 (0.33, 0.56)***	1.10 (0.73, 1.66)	5.58 (3.87, 8.07)***	1.56 (1.17, 2.07)**	0.83 (0.63, 1.11)	0.72 (0.48, 1.10)
Trinidad and Tobago	1.45 (1.20, 1.74)***	0.49 (0.31, 0.80)**	4.34 (2.67, 7.05)***	5.26 (3.32, 8.33)***	1.63 (1.00, 2.64)*	0.45 (0.27, 0.77)**	0.86 (0.40, 1.84)
United Arab Emirates	1.09 (0.94, 1.26)	0.48 (0.34, 0.68)***	1.33 (0.73, 2.42)	4.39 (3.17, 6.07)***	1.45 (1.09, 1.93)*	0.72 (0.48, 1.07)	0.97 (0.73, 1.30)
Uruguay	1.24 (0.99, 1.56)	0.39 (0.24, 0.62)***	2.14 (0.61, 7.55)	7.44 (4.18, 13.25)***	2.58 (1.35, 4.94)**	0.70 (0.39, 1.25)	1.12 (0.66, 1.89)
Overall	1.10 (1.06, 1.14)	0.63 (0.56, 0.72)	1.87 (1.67, 2.09)	5.55 (4.95, 6.21)	1.61 (1.52, 1.71)	0.95 (0.87, 1.03)	1.05 (0.97, 1.12)
I^2(p value)	32.2% (p=0.031)	74.6% (p=0.000)	40.3% (p=0.006)	67.6% (p=0.000)	24.7% (p=0.087)	50.0% (p=0.000)	29.3% (p=0.049)

$^{*}P<0.05$, $^{**}P<0.01$, $^{***}P<0.001$

AOR=Adjusted Odds Ratio; CI=Confidence Interval.

[a]Felt lonely most of the time or always in the past 12 months.

[b]Were physically attacked at least one time in the past 12 months.

[c]Parents understood their problems and worries, or know what they were doing with their free time most of the time or always in the past 30 days.

[d]Most of the students in their school were kind and helpful most of the time or always in the past 30 days.

[e]Occupied Palestinian territory.
